# Decoding the transcriptomic expression and genomic methylation patterns in the tendon proper and its peritenon region in the aging horse

**DOI:** 10.1186/s13104-023-06562-1

**Published:** 2023-10-11

**Authors:** Monica Y. Pechanec, Michael J. Mienaltowski

**Affiliations:** Department of Animal Science, University of California Davis, 2251 Meyer Hall, One Shields Ave, Davis, CA 95616 USA

**Keywords:** Equine, Tendon, Peritenon, RNASeq, RRBS, Bioinformatics

## Abstract

**Objectives:**

Equine tendinopathies are challenging because of the poor healing capacity of tendons commonly resulting in high re-injury rates. Within the tendon, different regions – tendon proper (TP) and peritenon (PERI) – contribute to the tendon matrix in differing capacities during injury and aging. Aged tendons have decreased repair potential; the underlying transcriptional and epigenetic changes that occur in the TP and PERI regions are not well understood. The objective of this study was to assess TP and PERI regional differences in adolescent, midlife, and geriatric horses using RNA sequencing and DNA methylation techniques.

**Results:**

Differences existed between TP and PERI regions of equine superficial digital flexor tendons by age as evidenced by RNASeq and DNA methylation. Cluster analysis indicated that regional distinctions existed regardless of age. Genes such as *DCN, COMP, FN1*, and *LOX* maintained elevated TP expression while genes such as *GSN* and *AHNAK* were abundant in PERI. Increased gene activity was present in adolescent and geriatric populations but decreased during midlife. Regional differences in DNA methylation were also noted. Notably, when evaluating all ages of TP against PERI, five genes (*HAND2, CHD9, RASL11B, ADGRD1*, and *COL14A1*) had regions of differential methylation as well as differential gene expression.

**Supplementary Information:**

The online version contains supplementary material available at 10.1186/s13104-023-06562-1.

## Introduction

Equine tendinopathies are an ongoing challenge due to the inadequate healing capacity of tendons, potential for incomplete repair and subsequent increased re-injury rates [1–4]. Tendon injuries represent almost 50% of musculoskeletal injuries in horses, and re-injury of recovered tendons like the superficial digital flexor tendon recur at least 50% of the time within three years [2, 5]. Moreover, aging significantly affects the overall outcome of tendon homeostasis and repair due to decreased tenocyte proliferation, increased matrix degeneration, and impaired structure at the extracellular, collagen, and gene expression levels [6–13]. Although it is well-established that tendon healing decreases with age, as seen in mechanical and age-related injury model studies, the biological processes and molecular functions underlying aging in equine tendon are not well understood [9, 12, 14]. Additionally, epigenetic changes associated with aging could contribute to impaired tendon healing, but limited studies have considered this process in aging tendon [15, 16]. Moreover, it is understood that differences exist in the contribution of the tendon proper (TP) and peritenon (PERI) regions of tendons to repair, particularly in immature versus mature tendons [7, 17–20]. Transcriptomic and epigenetic assessments of tendon aging within both TP and PERI regions could provide essential biological context for predicting how tendon cells might respond to therapeutics applied to injured equine tendons. The goals of this study were to elucidate gene expression markers defining the TP and PERI regions and those markers associated with maturation and aging, and to determine associations between DNA methylation and marker expression changes by age and location in the tendon.

## Methods

### Tendon harvest

Superficial digital flexor tendons (SDFTs) were harvested from fourteen thoroughbred horses from three age groups (0–5 years, adolescent; 6–14 years, midlife; and 15–23 years, geriatric) and two tendon regions (TP and PERI) (Table S1). Samples were collected from horses that were euthanized for reasons unrelated to this study; thus, they were exempt from approval of the University of California Davis Institute of Animal Care and Use Committee. The horses had no signs or known history of tendinopathies. TP tissue was isolated by excising a 2-2.5 mm diameter cylinder from the center core of the tendon. PERI tissue was isolated by harvesting some of the viscous paratenon in addition to 1 mm of the epitenon from the tendon. Samples were snap-frozen and powdered in liquid nitrogen and stored at -80 °C. A full description of tendon harvesting is provided in the Supplemental Material.

### RNA isolation, sequencing, RNAseq analysis, and RT-qPCR validation

TP and PERI samples were homogenized in QIAzol lysis reagent (Table S1). Total RNA was isolated with a QIAGEN RNeasy Plus Micro Kit following kit instructions with an on-column RNase-free DNase treatment (QIAGEN) [18]. RNA integrity was assessed via UV spectrophotometer (Nanodrop) and Experion Automated Electrophoresis Station (Bio-Rad). Total RNA with UV260:280 ratio > 1.9 and RNA integrity numbers > 8 were used. Total RNA from three horses per group was submitted to the UC Davis Genome Center for stranded library preparation (200–300 bp inserts) using TruSeq Stranded mRNA Sample Preparation kit (Illumina), barcoding, and sequencing done by Illumina HiSeqv4000 on two lanes to produce 150 bp pair-ended reads. A full description of the RNAseq analysis can be found in the Supplemental Material (Figure S1). Real-time quantitative polymerase chain reaction (RT-qPCR) assays were performed for target genes (Table S2) on TP and PERI tissues for 13 horses (Table S1) as described in the Supplemental Material.

### DNA isolation, RRBS, and DNA methylation analysis

DNA was isolated from TP and PERI tissues for ten horses (3 adolescent, 4 midlife, and 3 geriatric, Table S1) using the QIAamp Fast DNA Tissue Kit; DNA integrity was assessed by UV spectrophotometer (Nanodrop; UV260:280 > 1.8) and gel electrophoresis. Samples were submitted to the UC Davis Genome Center for preparation. Genomic DNA underwent restriction enzyme digestion with *MspI* to generate libraries enriched for CpG island and CpG shore regions. Adapters were added; then bisulfite conversion, barcoding, amplification, and purification were done. Samples were sequenced by an Illumina HiSeqv4000 to produce 100 bp single end reads. For 10x coverage, 10 samples per lane were loaded over 2 lanes for a coverage of 1.7 million CpGs [21]. Reduced-Representation Bisulfite Sequencing (RRBS) was used to assess CpG island regions throughout the genome [21]. A full description of the RRBS analysis can be found in the Supplemental Material (Figure S1).

## Results

### Transcriptomics

**Fig. 1:**
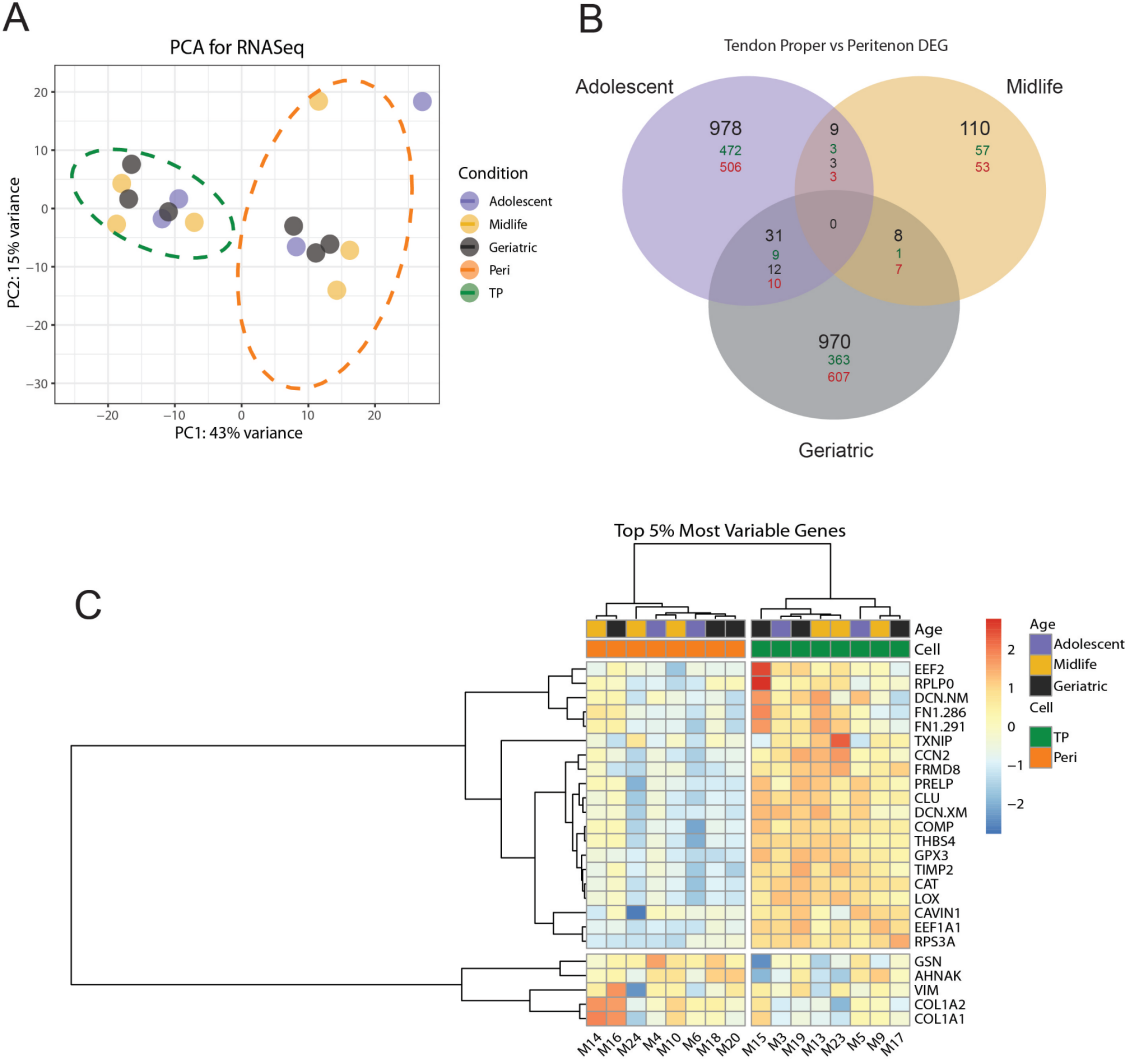
RNASeq analysis identifying sample relatedness. (**A**) The principal component analysis of all samples shows clear separation of TP and PERI tissue regions with limited separation of samples by age. (**B**) A Venn diagram of samples by age when comparing TP vs. PERI presented more DEGs in adolescent and geriatric ages. Black numbers signify the total DEGs, green as more abundant in TP compared to PERI, red as PERI more abundant than in TP, and blue as dependent on the sample the gene was either increased in TP or PERI. (**C**) A heatmap of the top 25 most variable genes further supported a separation between TP and PERI regions in genes closely tied to either region

Of the samples from the nine horses – three per age group – used for transcriptomics, eight samples were used for TP vs. PERI comparisons because Horse 25 proved to be an outlier as quantified using the Hubert Robust Principal Component Analysis Outlier (ROBPCA) computation and was excluded from further analysis (Figure S2) [22, 23]. Principal component analysis (PCA) of remaining samples demonstrated clear separation of the TP and PERI samples, with the TP samples clustering closer together compared to the PERI, and with heterogeneity in each cluster regarding sample age (Fig. 1A). For differentially expressed genes (DEGs), adolescent and geriatric age groups had far more (978; 970, respectively) DEGs compared to the midlife group (110) (Fig. 1B). Ratios of up- and down-regulated genes for the adolescent and midlife groups were about even; however, for the geriatric group, there were more down-regulated genes compared to the other two age groups. 446 DEGs were calculated when comparing TP and PERI for all eight horses. The top 25 most variable genes were plotted as a heatmap (Fig. 1C), which helps demonstrate the within-region (TP or PERI) heterogeneity in the two clusters of TP and PERI samples see in the PCA in Fig. 1A. Further distinctions between TP and PERI regions were seen regardless of age. Twenty-one of those twenty-five genes were upregulated in TP samples across all age groups; four genes were more abundant in PERI (Table S3).

**Fig. 2:**
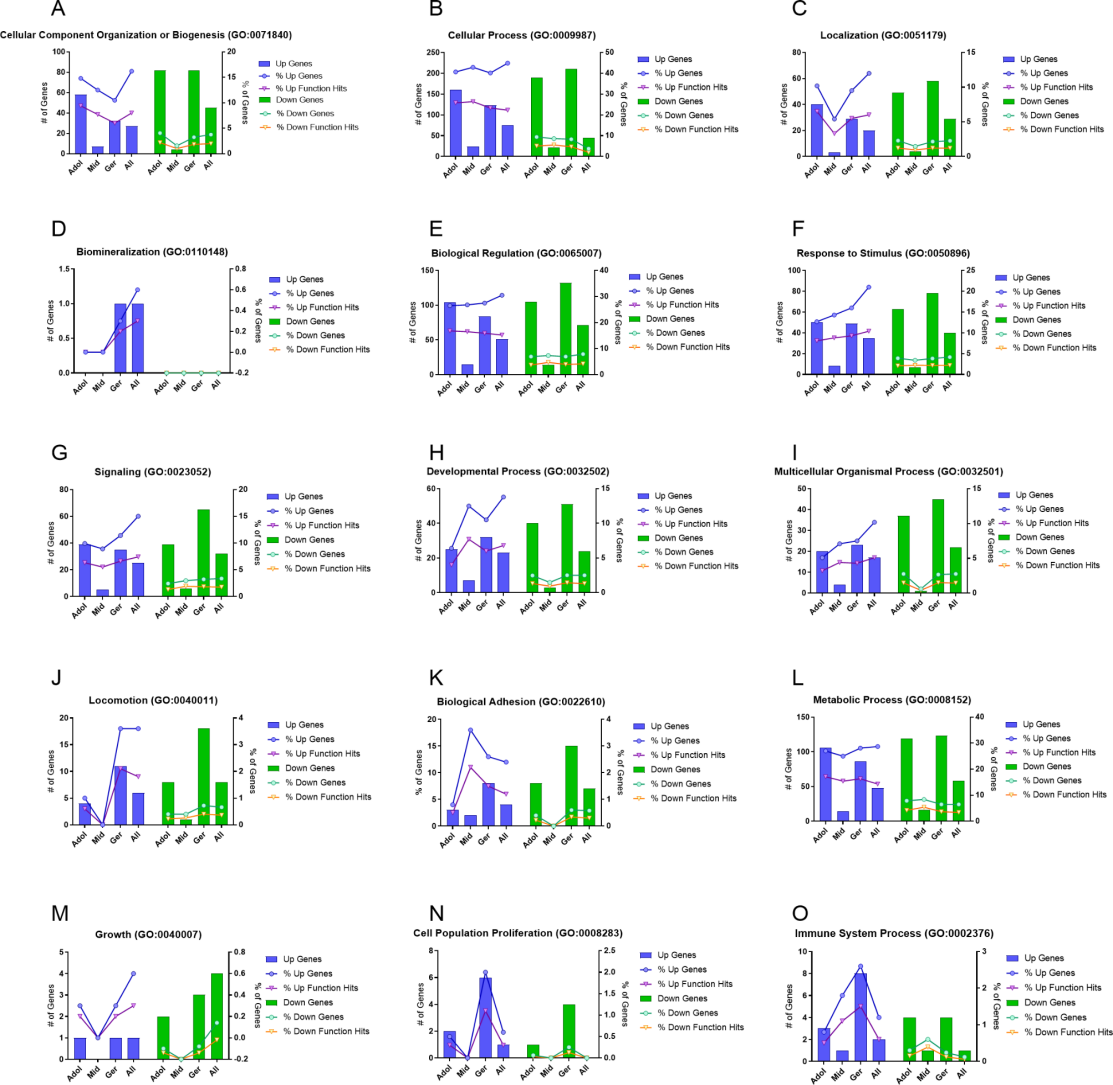
GO analysis for biological processes using PANTHER. Gene Ontology (GO) analysis for age groups between TP and PERI samples with at least two groups sharing a common GO term. Biological process terms are listed: (**A**) cellular component organization or biogenesis, (**B**) cellular process, (**C**) localization, (**D**) biomineralization, (**E**) biological regulation, (**F**) response to stimulus, (**G**) signaling, (**H**) developmental process, (**I**) multicellular organismal process, (**J**) locomotion, (**K**) biological adhesion, (**L**) metabolic process, (**M**) growth, (**N**) cell population proliferation, and (**O**) immune system process. Up regulated genes are in blue histogram bars while down regulated genes are in green. Further clarification for comparison was made with line graphs showing the percent of genes hit compared to the total genes (circle point line) and the percent of genes hit compared to the individual process (triangle point line)

PANTHER was used to analyze gene ontology (GO) for the TP vs. PERI comparisons. Fifteen biological process GO terms were shared between all age groups (Fig. 2). Fewer expressional differences were observed between TP and PERI regions in midlife horses when compared to adolescent and geriatric (Fig. 2). Notably, biological processes “cellular component organization or biogenesis” decreased from adolescent through midlife to geriatric while “response to stimulus” and “immune system process” increased between TP and PERI cells (Fig. 2A F, 2O). Moreover, “biomineralization” was only seen in aged samples, upregulated by TP; this trend persisted when considering all horses (Fig. 2D). Molecular function pathway analysis followed the same trend as for biological processes for both up and down regulated DEGs (Figure S3).

Tendon markers were also examined for these TP vs. PERI contrasts (Table S4). No differences were seen for midlife samples; however, adolescent and geriatric groups demonstrated changes for both PERI and TP. For example, in the geriatric group, numerous extracellular, organizational, and perivascular genes (*COMP, DCN, LOX, MKX*, and *CSPG4*) were significantly upregulated in the TP samples, as were genes known to increase mineralization (*BMP2*). *CD44* was downregulated in the geriatric group, further indicating the potential aging response that is occurring within the tendon between the TP and PERI. Expression patterns from transcriptomics were verified with RT-qPCR in Figures S4 and S5, with Figure S6 considering expression by sex.

### DNA methylation

**Fig. 3:**
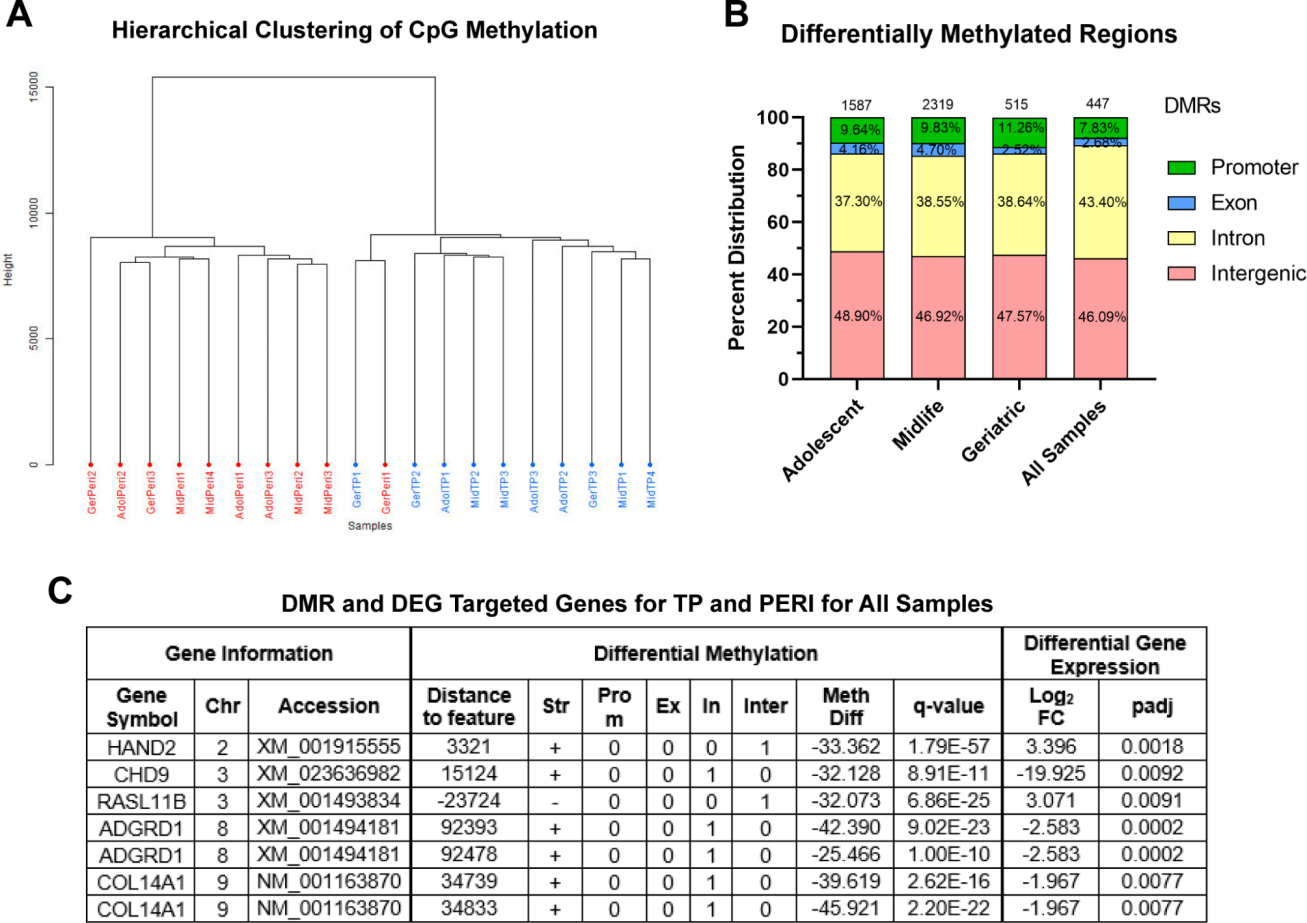
RRBS findings comparing methylation in TP and PERI regions. (**A**) CpG methylation clustering for all samples across all age groups. Hierarchical clustering of all samples showed separation of TP (blue) and PERI (red) tissue regions. A geriatric sample clustered with itself, indicating a potential underlying epigenetic alteration. There was no distinct separation between age groups (Adol, adolescent; Mid, midlife; Ger, geriatric). (**B**) Differentially methylated regions were present in the promoter, exon, and intergenic regions. Genomic regions were significant targets if the differential methylation was a q-value < 0.01 and differentially methylated > 25%. Annotation of gene regions was done to EquCab3.0 and promoter boundary flanking regions were > or < 2 kb of the transcription start site. (**C**) Five genes demonstrated both differential gene expression and differential methylation when comparing TP and PERI for all samples; these targets are hypomethylated in tendon proper samples and present in either the intron or intergenic regions. Two genes (*ADGRD1* and *COL14A1*) had two significantly differentially methylated regions associated with the gene target. Chr: chromosome; Str: strand; Prom: promoter; Ex: exon; In: intron; Inter: intergenic; Meth Diff, Differential Methylation

Cluster analysis for CpG methylation showed grouping emerging with TP and PERI (Fig. 3A), regardless of age, separated with a third group of a geriatric sample clustering with itself – perhaps the result of some underlying epigenetic factors present in that horse that could not be seen on a gross inspection or in gene expression. Further investigation of the specific genomic regions between TP and PERI populations shows differentially methylated regions (DMRs) present in all age groups. DMRs were annotated to the EquCab3.0 genome for distances to transcription start sites (TSS), promoter, exon, intron, and intergenic regions, and association to gene features. Total DMRs ranged from 2319 in midlife to 447 in all samples (515 geriatric; 1587 adolescent) (Fig. 3B). DMRs were found from greatest to least in the following regions: intergenic, intron, promoter, and exon, respectively (Fig. 3B). The methylation changes between the TP and PERI regions in all horses revealed a greater proportion of hypomethylation across all chromosomes for TP with cutoff values of q-value < 0.01 and percent methylation difference > 25% (Figure S7).

When comparing DMRs and DEGs for TP vs. PERI contrasts for all horses, 8 differential expression and methylation targets were identified across 5 genes (*ADGRD1, CHD9, COL14A1*, *HAND2*, and *RASL11B*) (Fig. 3C). Gene targets were mainly located within introns, except for *HAND2* and *RASL11B*, which were in intergenic regions. All target genes were hypomethylated in TP, or conversely hypermethylated in PERI regions.

## Discussion

Previous studies demonstrated physiological differences between TP and PERI cells of tendon [17, 18, 24]. We too found regional physiological genomic differences, namely differential gene expression and differentially methylated regions. Regardless of age, common tendon genes like *DCN, COMP*, and *LOX* maintained elevated expression in TP while genes such as *GSN*, a cell migration, proliferation, and inflammatory gene, and *AHNAK*, a cell proliferation and differentiation gene, were more abundant in PERI [25]. Those PERI-abundant genes could contribute to the cells’ role in early migration, proliferation, and inflammatory response that accompanies injuries [19, 26–28], while TP-abundant genes corroborate essential expression from the TP as it supplies proteins essential for maintenance and upkeep of extracellular matrix (ECM), which is of particular importance for preserving biomechanical properties of tendons in aging and repair [10, 29–34].

When evaluating DMRs and DEGs across all ages to compare between TP and PERI, five genes were identified. Transcripts for three genes were greater in PERI (*CHD9, ADGRD1*, and *COL14A1*), while two genes were for TP (*HAND2* and *RASL11B*). The adhesion G protein-coupled receptor (GPCR) family (*ADGRD1*) plays pivotal roles in the musculoskeletal system, though roles in tendon have yet to be elucidated [35, 36]. Additionally, *CHD9* has been implicated in upregulating *RUNX2* which may shed light on tendinopathy-associated ectopic ossification [37]. The protein encoded by *COL14A1* has been show to regulate collagen fibrillogenesis in tendon development and early post-natal life; a recent study has localized *COL14A1* transcripts in cells along the edge of medial collateral and anterior cruciate ligaments including two sub-populations of COL14A1-positive ligament fibroblasts with either important cell migration and pro-angiogenesis signatures or highly favorable stemness signatures, respectively [38, 39]. With pro-migration and angiogenesis features, such fibroblasts could be the cells initially active in tendon repair, which agrees with previous studies [19, 26, 27]. *HAND2* plays a major role in limb development, is crucial for the establishment of the anterior-posterior axis but is also required for vascular development and angiogenesis regulation; perhaps *HAND2* expression in TP is a result of its proximity to the vascularization from PERI, though it could also be expressed residually in the equine SDFT cells as part of maintaining a cell’s fate in the posterior distal limb [40–45]. The *RASL11B* gene was more abundant in TP; it plays a role in maturation of primary macrophages which can affect TGF-B1-mediated developmental and inflammatory processes and has been observed to promote ERK1/2 and SMAD2/3 signaling in the presence of hyaluronic acid for amniotic stem cells induced into chondrogenesis [46, 47]. Given the role of ERK1/2 and SMAD signaling to activate Scleraxis and Mohawk in tendon development, RASL11B could play a similar role for tenogenic differentiation [48]. Thus, while more needs to be elucidated about the roles of these genes, their particular expression and methylation signatures provide us with targets to consider in regard to stemness, cell migration, angiogenesis, and ectopic ossification in TP and PERI regions of the SDFT.

Notably, the greatest gene variation occurred in the adolescent and geriatric stages of a horse’s life. In adolescence, the differentially expressed genes between TP and PERI were similar in amount; however, in the geriatric tendon, expression shifts were downward for TP and upward for the PERI region. Given the decreased capacity of the tendon proper to maintain ECM homeostasis with age – which was even seen with increased biomineralization activity in geriatric TP – these expression findings could suggest the increased activity of PERI cells to compensate [49–51]. Such compensation is further corroborated with the increases in ECM matrix assembly (*COMP*, *DCN, LOX, MKX*) and vascular (*CSPG4*) markers with decreased *CD44* expression, further supporting that even in uninjured tendon, at a geriatric age, the tendon is already undergoing some repair process as a result of compositional changes [52–55]. Contrastingly, during midlife DEGs were minimal between TP and PERI; all of our analyses indicated that there was a level of homeostasis occurring with TP and PERI at that stage of life. Growth, development, and maturation occur with adolescence; responses to degenerating structure ensue in the geriatric age. We found that DMRs in midlife were much greater compared to the other groups. Still, a majority of the DMRs represented hypomethylation in TP relative to PERI, which indicated that expressional differences and their lack thereof could be related to factors outside of control by epigenetic factors. Furthermore, this general overall similarity (or lack of defined difference) in expression between TP and PERI regions in mid-life coinciding with less methylation of TP cells’ genomes suggest that cells in the TP region could be equally responsive to the same stimuli or interventions; however, less epigenetic regulation in the TP cells could lead to relaxed regulation of gene expression, increased yet perhaps aberrant gene expression, and more potential for dysdifferentiation of TP cells, perhaps affecting proper healing and leading to injury recurrence [56–58].

### Limitations

Some limitations to this study include the limited number of equine samples used for RNASeq and DNA methylation analysis, which makes it difficult to assess breed, sex, and athletic performance differences, for example. Additionally, we did not segregate out the several cell populations that could contribute to physiology in these unique tendon regions; thus several cell types are contributing to the tissue phenotyping we described. Furthermore, although no known tendinopathies were present in the horses in the study, full histories of exercise regimens were not provided.

### Electronic supplementary material

Below is the link to the electronic supplementary material.

Supplementary Material 1

## Data Availability

The datasets for this study are available in the NCBI Short Read Archive repository under accessions BioProject PRJNA802905 (RNAseq) and BioProject PRJNA803631 (RRBS).

## List of abbreviations

ADGRD1: Adhesion G Protein-Coupled Receptor D1
AHNAK: Neuroblast Differentiation-Associated Protein AHNAK, Desmoyokin
BMP2: Bone Morphogenic Protein 2
CD44: Cell-Surface glycoprotein, Homing Cell Adhesion Molecule
cDNA: Complementary Deoxyribonucleic Acid
CHD9: Chromodomain Helicase DNA Binding Protein 9
COL14A1: Collagen Type XIV alpha I
COL1A1: Collagen Type I alpha I
COL1A2: Collagen Type I alpha II
COMP: Cartilage Oligomeric Matrix Protein
CSPG4: Chondroitin Sulfate Proteoglycan 4
DCN: Decorin
DEG: Differentially Expressed Gene
DMR: Differentially Methylated Region
DNA: Deoxyribonucleic Acid
ECM: Extracellular Matrix
EMCN: Endomucin
ERK1/2: Extracellular Signal‑Regulated Protein Kinase
FDR: False Discovery Rate
GO: Gene Ontology
GPCR: G Protein-Coupled Receptor
GSN: Gelsolin
HA: Hyaluronic Acid
HAND2: Heart and Neutal Crest Derivatives Expressed 2
LFC: Log fold change
LOX: Lysyl Oxidase
MKX: Mohawk
PANTHER: Protein ANalysis THrough Evolutionary Relationships
PCA: Principal Component Analysis
PERI: Peritenon
RASL11B: RAS Like Family 11 Member B
RNA: Ribonucleic Acid
RNASeq: RNA Sequencing
ROBPCA: Hubert Robust Principal Component Analysis Outlier
RRBS: Reduced-representation Bisulfate Sequencing
RUNX2: Runt-Related Transcription Factor 2
SDFT: Superficial Digital Flexor Tendon
SMAD: Mothers Against Decapentaplegic Pathway
TGFB1: Transforming Growth Factor Beta 1
TP: Tendon Proper
TSS: Transcription Start Site
VST: Variance Stabilizing Transformation
