# Supplementary material for: Decoding the transcriptomic expression and genomic methylation patterns in the tendon proper and its peritenon region in the aging horse
Source: BMC Res Notes. 2023 Oct 11;16:267. doi: 10.1186/s13104-023-06562-1 (PMC10566085; doi:10.1186/s13104-023-06562-1)
Supplement: Supplementary file 1 — Supplementary Material 1 [file 13104_2023_6562_MOESM1_ESM.pdf]

Supplementary Materials

Supplemental Methods

Table S1. Sample Details

Horse	Age	Breed	Sex	RNAseq (IDs)	RRBS	RT-qPCR
1	6 y	TB	Male			X
6	12 y	TB	Female	X (7,8)	X	X
7	14 y	TB	Male	X (17,18)	X	X
8	17 y	TB	Male	X (11,12)	X	X
10	12 y	TB	Male		X	X
11	22 y	TB	Gelding			X
13	23 y	TB	Gelding	X (13,14)	X	X
14	9 y	TB	Female	X (9,10)	X	X
15	5 y	TB	Female	X (1,2)	X	X
16	5 y	TB	Female	X (3,4)	X	X
20	21 y	TB	Female	X (15,16)	X	
23	13 y	TB	Gelding			X
25	10 d	TB	Female	X (5,6)	X	X
27	1 mo	TB	Male			X

TB, Thoroughbred; RNAseq IDs given are referenced in Figure S3.

Tendon Harvest

Superficial digital flexor tendons were harvested from Thoroughbred horses. Superficial digital flexor tendon (SDFT) samples were collected 10-15 cm proximal to the forelimb fetlock were harvested, rinsed a minimum of three times in fresh Dulbecco's Phosphate Buffer Solution (Life Technologies) containing 1% antibiotic/antimycotic (10,000 units/mL penicillin, 10,000 ug/mL streptomycin, and 25 ug/mL amphotericin B, Life Technologies) before transport [1, 2]. Samples were either immediately snap frozen in liquid nitrogen as whole tendons or isolated into the tendon proper and peritenon regions under a dissecting microscope, snap frozen, and then stored at -80° C until further processed. TP tissue was isolated by excising a 2-2.5 mm diameter cylinder from the center core of the tendon. PERI tissue was isolated by harvesting some of the viscous paratenon in addition to 1 mm of the epitenon from the tendon. Samples were sectioned, if not already, on dry ice and powdered while frozen to allow for better RNA and DNA isolation.

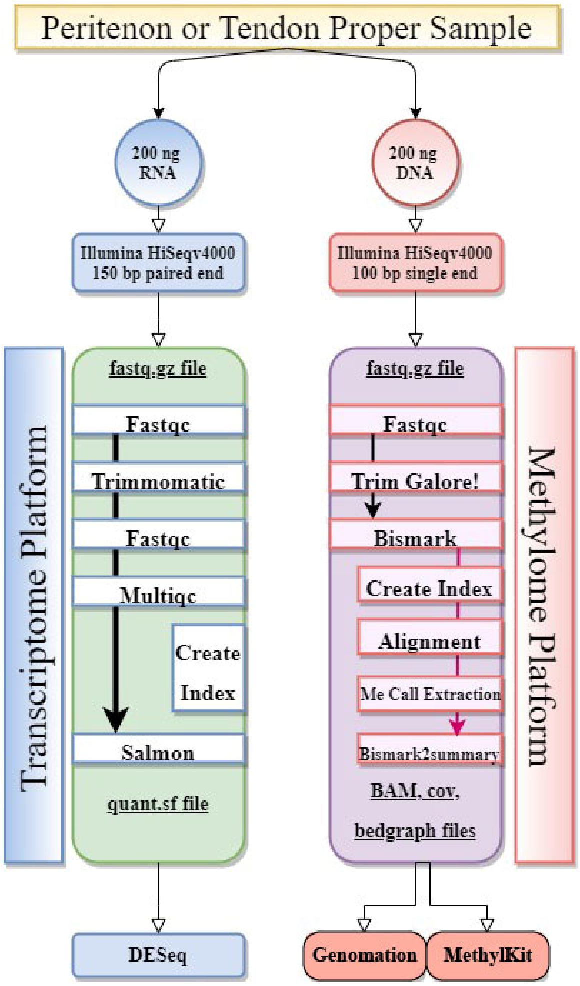

Figure S1. The workflow for sample preparation and analysis. Samples were taken through either the transcriptome or methylome workflow platform with alignment to EquCab3.0 before further downstream analysis.

RNAseq Analysis

RNASeq analysis was conducted using the Ubuntu 18.04.2 LTS with miniconda2 for package management and downstream analysis in RStudio v 1.4.463 [3-6]. Untrimmed FASTQ files were checked for corruption using respective md5sum files and then quality controlled using FastQC 0.11.8 [7]. All samples showed high quality and thus progressed to adapter trimming by Trimmomatic 0.39 (PE; Illuminaclip: TruSeq2-PE.fa:2:0:15;

LEADING:15; TRAILING:15; SLIDINGWINDOW:10:20; MINLEN:25) and were reexamined for quality controlled using FastQC and MultiQC 1.7 [8, 9]. Transcriptome mapping was accomplished using Salmon 0.14.1 and mapped to the horse genome (EquCab3.0) to produce quantification files to be further processed in RStudio using DESeq2 1.30.0 [10-15] **(Figure S1)**. A mean of 17.2 million read pairs were generated per sample with a mean 62.2% kept for alignment to the genome after QC and removing redundant reads. Samples in DESeq2 were subset into age groups (Adolescent, Midlife, Geriatric) and contrasted for cell type (TP vs PERI); in addition, one analysis contained all samples with a cell type contrast using a Wald significance test. Differentially expressed genes (DEGs) were considered as genes that had a q value (false discovery rate; FDR) < 0.01 and a log 2-fold change (L2FC) < -1 and > 1. Additionally, a variance stabilizing transformation (vst) was performed for Principal Component Analysis (PCA) and the top 25 most variable genes for heatmapping which utilized clustering rows and columns by Pearson correlation and the method by Ward.D. Gene Ontology (GO) was accomplished using PANTHER from differentially expressed genes (DEGs) hits [16].

DNA Methylation Analysis

Output FASTQ files from the Illumina HiSeqv4000 sequencer with a mean of 15.9 million reads per sample were first quality controlled using FastQC before undergoing adapter trimming [14, 15]. FASTQ files were adapter trimmed using Trim Galore! 0.6.5 and then processed using Bismark 0.20.0 with mapping to EquCab3.0 [11, 14, 15, 17, 18]. Bismark was used for genome preparation, alignment, and methyl call extraction to generate coverage and BAM files to be used in further analysis. Methylkit 1.16.0 was

used for generation of methyl calls, CpG islands, and genomic annotation [19] **(Figure S1)**. Samples were filtered by read coverage to prevent PCR bias and increase the power of the statistical tests by discarding bases with high (above 99.9th percentile) and low (below 10x) read coverage with each sequenced and filtered CpG site assigned a percentage methylation score. Systematic over-sampling bias potentially affecting statistical tests was reduced by normalizing coverage. Hierarchical clustering used Canberra distances and subsequently Ward for the method. Annotation of differentially methylated regions was performed using the package Genomation 1.22.0 and referenced to EquCab3.0 with promoter regions designated at 2kb up and downstream of the transcription start site (TSS) [20].

RT-qPCR

Real-time quantitative polymerase chain reaction assays (RT-qPCR) were used to validate RNAseq findings. Briefly, for each TP and PERI tissue from thirteen horses, 500 ng total RNA was reverse transcribed into cDNA using a High Capacity cDNA Reverse Transcription Kit (Life Technologies) and applied to TaqMan assays for target genes with *POLR2A* as the normalizing gene **(Table S2)**, as well as Fast Advanced Mastermix (Life Technologies), on a StepOnePlus Real-Time PCR System (Applied Biosystems) [2, 21]. Gene specific efficiencies were calculated using LinRegPCR v7.5 software for each qPCR plate and the relative quantity of mRNA for each gene of interest was computed using the relative gene expression ratios formula [22-25]. Expression was analyzed with Prism (GraphPad Software, Inc.) by age group using mixed-effects model with Sidak's multiple comparisons test **(Figure S4)** and using simple linear regression of relative expression vs. age **(Figure S5)**.

Table S2. RT-qPCR Targets

Gene Symbol	TaqMan Assay ID
CHD9	Ec06989350_m1
COL1A1	Custom AIRS8KX
COMP	Ec03468073_m1
DCN	Custom AIFATUP
HAND2	Ec07037343_m1
LOX	Custom APWCYFZ
MKX	Custom AILJKPT
POLR2A	Custom APT2EKG
SCX	Ec03818452_s1

Supplemental Results

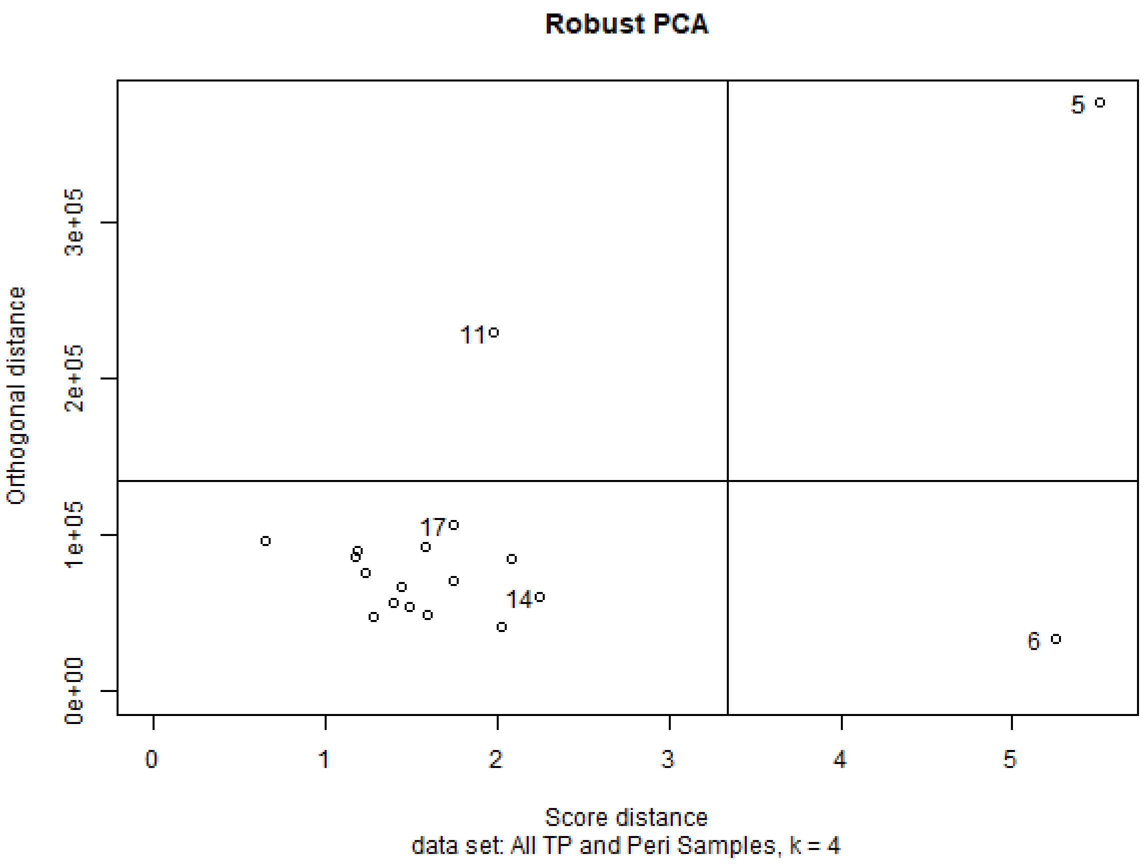

Figure S2. Robust PCA for Outlier Detection. A robust variation of outlier detection using the Hubert ROBPCA was done using normalized counts. Based on the screeplot generated for the normalized data of all the samples, k = 4 was determined as the number of principle components to retain. The 10-day old TP sample (denoted as 5 on the plot) was outside the Wilson-Hilferty transformation for a χs^2 distribution (orthogonal distance; y-axis) and the 97.5% quartile of χ^2_k distribution (score distance; x-axis). Samples 6 (PERI 10-day old) and 11 (TP geriatric) were outside one of the two criteria and therefore were not immediately excluded. The 10-day old TP Sample 5 was excluded with its Sample 6 PERI counterpart, representing Horse 25. Plotting for the outlier test was conducted using the PcaHubert command within the rrcov package.

Table S3. Top 25 most variably expressed genes.

Gene	RefSeq	Gene Name	Cell Type	Variable Abundance
AHNAK	XM_023654210	AHNAK nucleoprotein	PERI	-0.132
CAT	XM_001914718	Catalase	TP	1.935
CAVIN1	XM_001494473	Caveolae associated protein 1	TP	0.359
CCN2	XM_023651101	Cellular communication network factor 2	TP	1.212
CLU	NM_001081944	Clusterin	TP	1.180
COL1A1	XM_023652710	Collagen type I alpha I	PERI	-0.335
COL1A2	XM_001492939	Collagen type I alpha 2	PERI	-0.666
COMP	NM_001081856	Cartilage oligomeric matrix protein	TP	2.182
DCN.NM	NM_001081925	Decorin	TP	0.513
DCN.XM	XM_005606467	Decorin	TP	1.008
EEF1A1	NM_001081781	Eukaryotic translation elongation factor 1 alpha 1	TP	0.192
EEF2	XM_001915097	Eukaryotic translation elongation factor 2	TP	0.105
FN1.286	XM_023642286	Fibronectin 1	TP	0.595
FN1.291	XM_023642291	Fibronectin 1	TP	0.934
FRMD8	XM_023654435	FERM domain containing 8	TP	0.638
GPX3	NM_001115158	Glutathione peroxidase 3	TP	1.251
GSN	XM_023628604	Gelsolin	PERI	-0.420
LOX	XM_023617820	Lysyl oxidase	TP	1.625
PRELP	XM_001915274	Proline and Arginine rich end leucine rich repeat protein	TP	1.134
RPLP0	NM_001252576	Ribosomal protein lateral stalk subunit P0	TP	0.138
RPS3A	XM_001501474	Ribosomal protein S3A	TP	0.255
THBS4	XM_023618094	Thrombospondin 4	TP	1.622
TIMP2	XM_023651899	TIMP metallopeptidase inhibitor 2	TP	0.723
TXNIP	XM_023641298	Thioredoxin interacting protein	TP	0.259
VIM	NM_001243145	Vimentin	TP	0.006

Note: Transcript variants existed for *DCN* and *FN1*

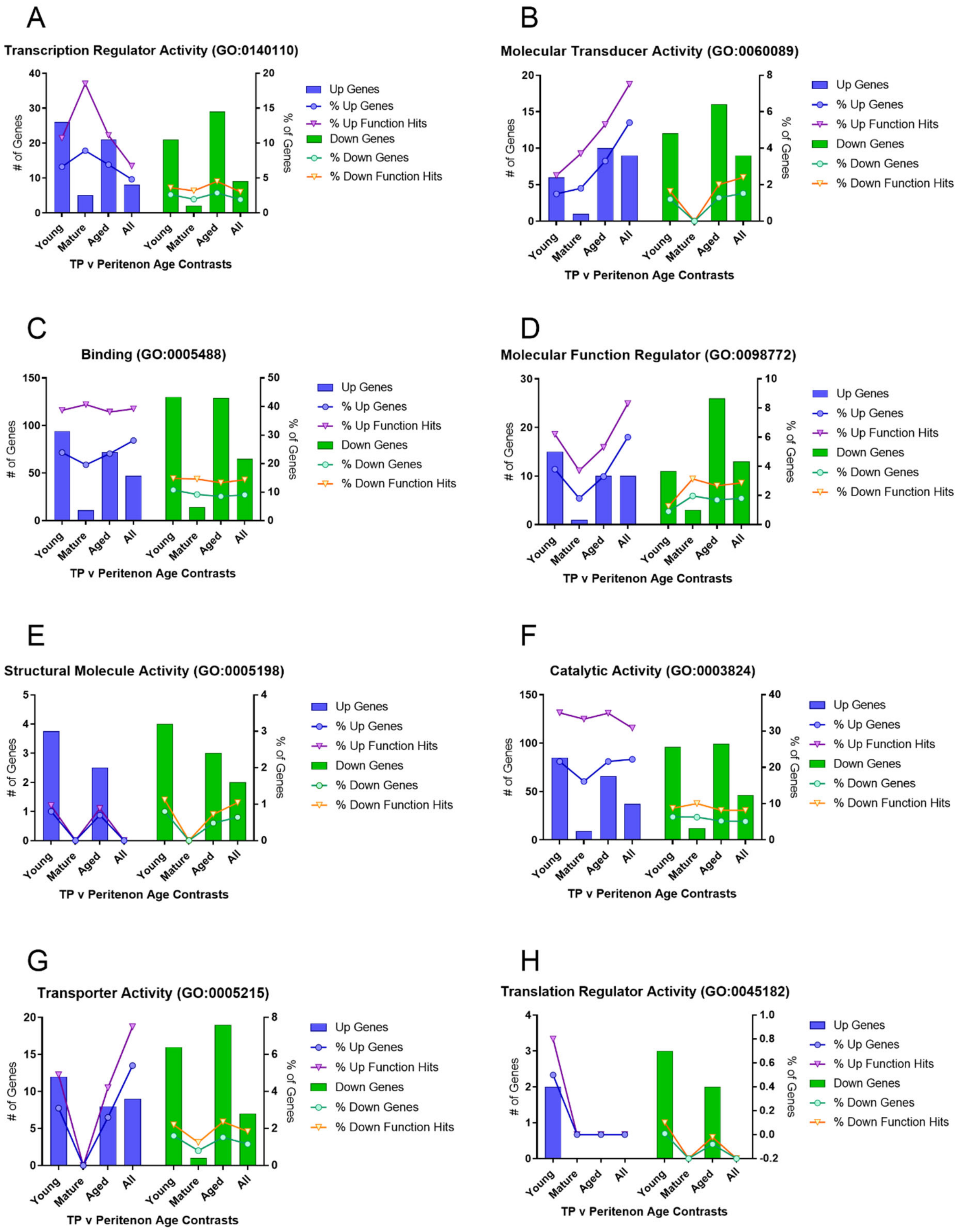

Figure S3. GO analysis for molecular function using PANTHER. Gene Ontology (GO) analysis for age groups between TP and PERI samples with at least two groups sharing

a common GO term. Molecular function terms are listed: **(A)** transcription regulator activity, **(B)** molecular transducer activity, **(C)** binding, **(D)** molecular function regulator, **(E)** structural molecule activity, **(F)** catalytic activity, **(G)** transporter activity, and **(H)** translation regulator activity. Upregulated genes are plotted as blue histogram bars and downregulated genes in green. Line graphs were used to further clarify comparisons with circle point lines showing the percent of genes hit to the total genes and the triangle point lines indicating the percent of genes hit for an individual function.

Table S4. RNASeq differential expression for tendon related genes of interest.

		Adolescent TP v PERI		Midlife TP v PERI		Geriatric TP v PERI		All TP v PERI	
Gene Symbol	RefSeq	padj	L2FC	padj	L2FC	padj	L2FC	padj	L2FC
BGN	NM_001081839	0.8510	-0.5706	0.99995	-1.3885	0.7059	1.0439	0.9998	0.2716
BMP1	XM_005607660	**0.000002**	**-1.7199**	0.99995	-0.7324	0.9796	-0.1753	0.9998	-0.0160
BMP1	XM_005607662	0.9999	-0.1805	0.99995	-0.9983	0.9166	0.8803	**0.0041**	**8.0417**
BMP2	XM_023626136	0.9999	-0.0311	0.99995	0.8360	**0.0002**	**1.9449**	**0.0064**	**1.9852**
CD44	XM_005598023	**0.0012**	**1.0756**	0.99995	-0.1368	**0.0399**	**-0.8104**	0.9998	-0.3052
COL1A1	XM_023652710	**0.0275**	**-1.1807**	0.99995	-0.6733	0.8201	-0.7596	0.9998	0.1383
COL1A2	XM_001492939	0.3906	-0.9936	0.99995	-0.6628	0.5029	-1.1818	0.9998	-0.5151
COL5A1	XM_023629284	**0.0133**	**-0.7507**	0.99995	-1.0524	0.9741	-0.1735	0.9998	-0.1165
COL14A1	NM_001163870	0.9755	0.4472	0.99995	-0.8647	**0.00090**	**-1.8458**	**0.0077**	**-1.9667**
COMP	NM_001081856	0.9918	-0.7957	0.99995	0.1852	**0.0018**	**3.4633**	**0.0162**	**4.5950**
CSPG4	XM_005602901	0.5524	-1.3342	0.99995	-0.2909	**0.0219**	**1.6050**	0.2564	1.9340
DCN	NM_001081925	0.9853	-0.3141	0.99995	-1.0096	0.3940	1.3663	0.6764	1.2716
DCN	XM_005606467	0.9875	0.4134	0.99995	-0.6091	**0.0120**	**1.9728**	**0.0426**	**2.2465**
EGR1	XM_001502553	0.1714	1.0839	0.99995	-0.0547	0.8385	0.3928	0.9471	-0.4547
EMCN	XM_014738644	**0.00007**	**-8.6756**	0.89027	7.3201	1.0000	-2.8830	0.9175	-0.4980
EMCN	XM_023637656	**0.0034**	**7.7222**	1.00000	-4.0162	0.9897	-0.2128	0.9998	0.6460
FMOD	NM_001081777	0.7095	-1.1449	0.99995	-1.7627	0.3612	1.6307	0.6198	1.7823
FMOD	XM_005609562	0.7049	-1.2166	0.99995	-1.5459	*0.0857*	*2.1297*	0.4892	1.9520
LOX	XM_023617820	0.9866	0.8119	0.99995	0.6449	**0.0064**	**2.6260**	**0.0017**	**3.6832**
LOX	XM_023617821	0.9999	-0.4132	0.99995	0.1023	**0.0155**	**2.9197**	**0.0007**	**4.7060**
MKX	XM_023632372	0.9999	0.2827	0.99995	0.9987	**0.0244**	**2.3604**	**0.0416**	**2.5332**
SCX	NM_001105150	0.4276	-2.3190	0.99995	0.4775	0.0626	2.8441	0.6767	2.1033
TIMP1	XM_023633181	0.9999	0.0389	0.99995	-0.6297	0.9972	0.0180	0.9998	0.0881
TIMP2	XM_023651899	0.9997	0.1975	0.99995	0.6356	0.1015	1.3328	0.0644	1.5320

Note: Gene contrasts with a false discovery rate (padj) < 0.01 are in bold with the accompanying log 2-fold change (L2FC) while gene contrasts between 0.1 and 0.05 padj are in italics. All genes have their accompanying RefSeq number specifying the gene variant. Significant gene contrasts that have a positive L2FC, indicating higher expression in tendon proper or a contrastingly lower expression in peritenon, are

highlighted in green while genes that have a negative L2FC, indicating higher expression in peritenon or contrastingly lower expression in tendon proper, are highlighted in orange.

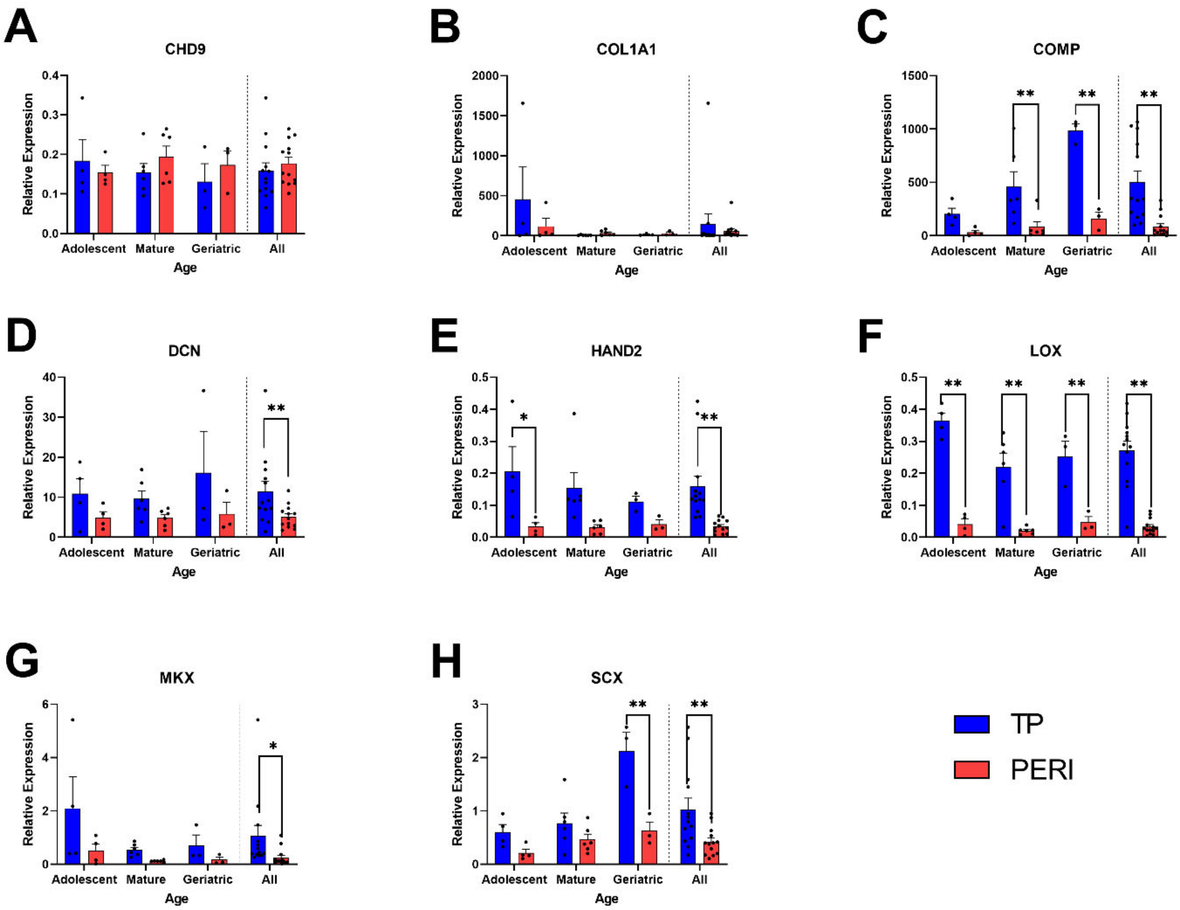

Figure S4. RT-qPCR of genes of interest to validate TP vs. PERI RNAseq comparisons by age group. DEGs from RNAseq data were validated with RT-qPCR. Genes evaluated included **(A)** *CHD9*, **(B)** *COL1A1*, **(C)** *COMP*, **(D)** *DCN*, **(E)** *HAND2*, **(F)** *LOX*, **(G)** *MKX*, and **(H)** *SCX*, relative to normalizing housekeeping gene *POLR2A*. Samples were grouped by age (adolescent, mature, geriatric) and a comparison for all samples was made. Significant differences in gene expression between TP and PERI regions were confirmed for *COMP* **(C)**, *DCN* **(D)**, *HAND2* **(E)**, *LOX* **(F)**, *MKX* **(G)**, and *SCX* **(H)**. Mixed-effects model with Sidak's multiple comparisons test were applied; * represents adjusted $p < 0.05$, and ** represents adjusted $p < 0.01$.

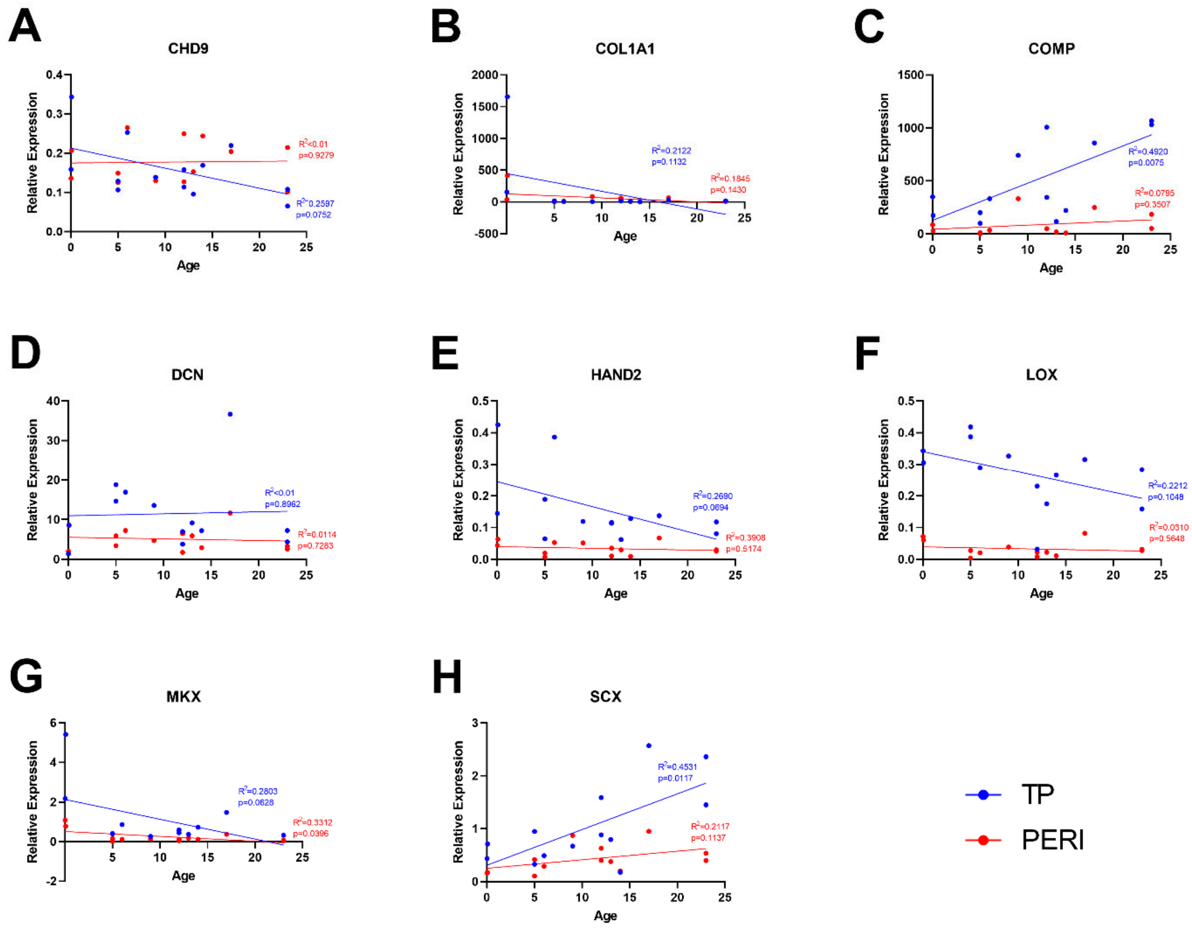

Figure S5. Linear regression analysis of RT-qPCR to survey gene expression by age. RT-qPCR findings for expression of genes **(A)** *CHD9*, **(B)** *COL1A1*, **(C)** *COMP*, **(D)** *DCN*, **(E)** *HAND2*, **(F)** *LOX*, **(G)** *MKX*, and **(H)** *SCX*, relative to normalizing housekeeping gene *POLR2A*, were also analyzed using linear regression comparing expression vs. age. Expression of *CHD9* **(A)**, *HAND2* **(E)**, *LOX* **(F)**, and *MKX* **(G)** trended downward with age for TP, though expression of *HAND2*, *LOX*, and *MKX* was greater for TP samples relative to PERI. Levels of *COMP* **(C)** and *SCX* **(H)** significantly increased over time for TP and were higher than PERI. Simple linear regression was applied, plotting each horse's expression vs. its age.

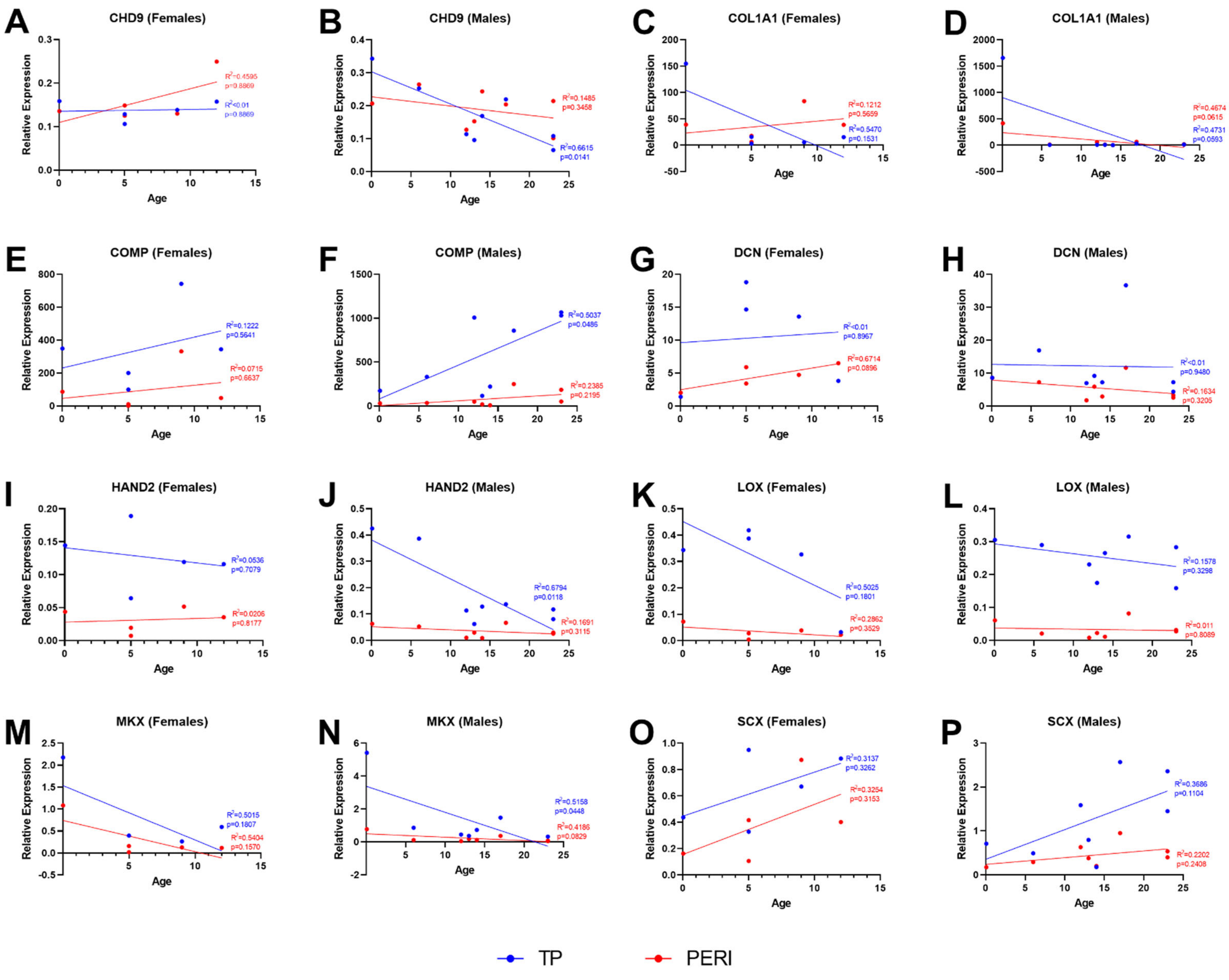
A
CHD9 (Females)
B
CHD9 (Males)
C
COL1A1 (Females)
D
COL1A1 (Males)
E
COMP (Females)
F
COMP (Males)
G
DCN (Females)
H
DCN (Males)
I
HAND2 (Females)
J
HAND2 (Males)
K
LOX (Females)
L
LOX (Males)
M
MKX (Females)
N
MKX (Males)
O
SCX (Females)
P
SCX (Males)
Relative Expression
Age
TP
PERI

Figure S6. Linear regression analysis of RT-qPCR to survey gene expression by age for each sex. RT-qPCR findings for expression of genes **(A,B)** *CHD9*, **(C,D)** *COL1A1*, **(E,F)** *COMP*, **(G,H)** *DCN*, **(I,J)** *HAND2*, **(K,L)** *LOX*, **(M,N)** *MKX*, and **(O,P)** *SCX*, relative to normalizing housekeeping gene *POLR2A*, were also analyzed using linear regression comparing expression vs. age. Significant decreases in expression with age were seen for *CHD9* for TP in males **(B)**, *HAND2* for TP in males **(J)**, and *MKX* for TP in males (**N**). Significant increases in expression with age were seen for *COMP* for TP in males **(F)**. Simple linear regression was applied, plotting each horse's expression vs. its age, considering sex.

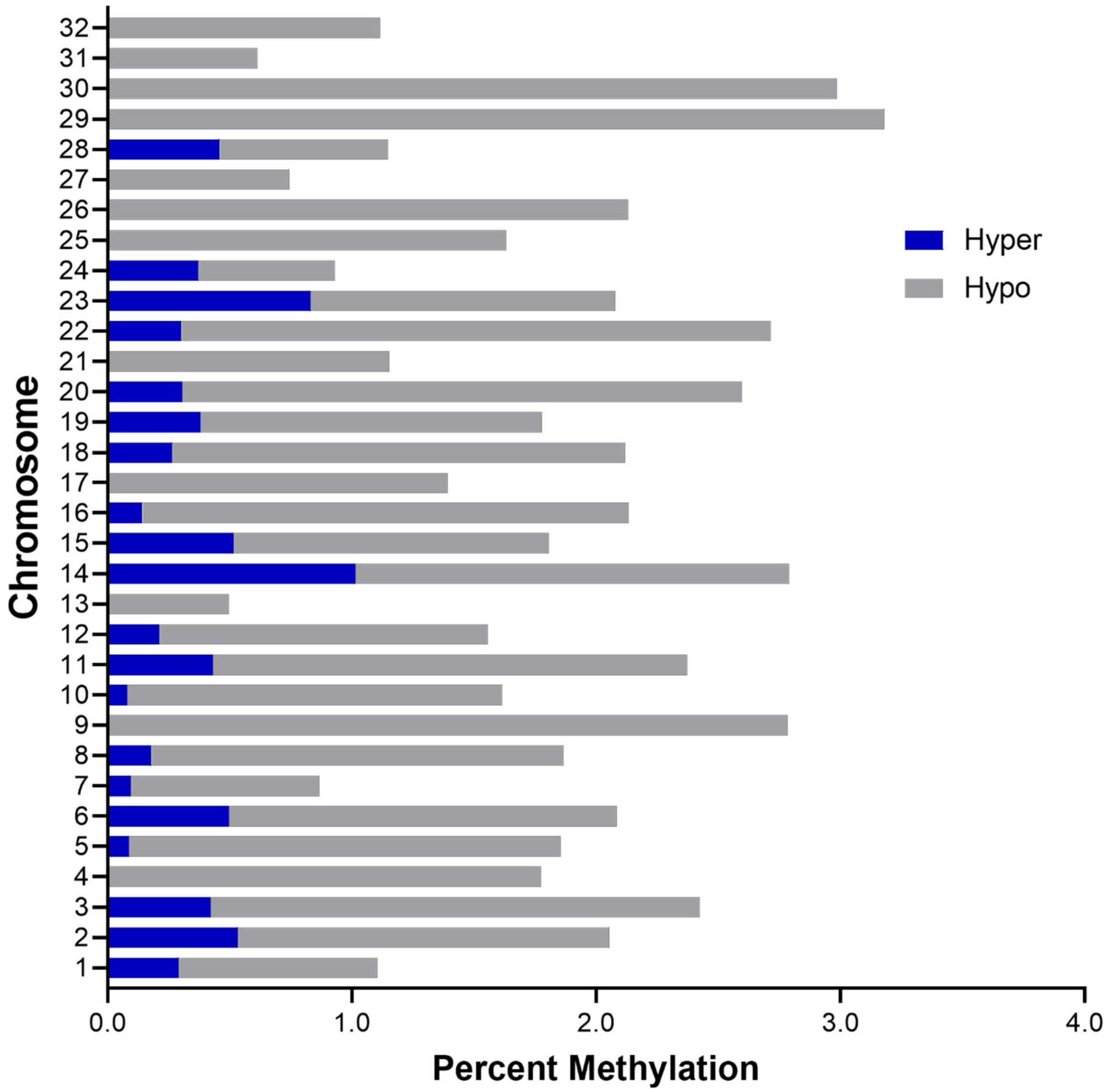

Figure S7. Percent methylation differences across all equine chromosomes when comparing TP against PERI for all samples. When looking at hyper- and hypomethylation, the tendon proper expresses more regions of hypomethylation across all chromosomes compared to the peritenon. The peritenon is treated as a 'control' and the tendon proper methylation calls are designated as 'treatment'. Although some increased regions of hypermethylation also occur, such as on Chr 14 and 23, substantially more chromosomes are hypomethylated in the tendon proper compared to the peritenon. Differentially methylated regions were considered hits with a q-value < 0.01 and percent differential methylation > 25% between groups.

Supplemental Materials References

[1] Mienaltowski MJ, Adams SM, Birk DE. Regional differences in stem cell/progenitor cell populations from the mouse Achilles tendon. *Tissue Eng Part A.* 2013; 19:199-210.

[2] Pechanec MY, Lee-Barthel A, Baar K, Mienaltowski MJ. Evaluation and Optimization of a Three-Dimensional Construct Model for Equine Superficial Digital Flexor Tendon. *J Equine Vet Sci.* 2018; 71: 90-97.

[3] Sobell MG. A practical guide to Ubuntu Linux. Pearson Education. 2015.

[4] Anaconda Software Distribution. *Miniconda2* [Computer software]. 2016. Retrieved from https://www.anaconda.com

[5] RStudio Team. RStudio: Integrated Development for R. RStudio, PBC, Boston, MA. 2020. Retrieved from http://www.rstudio.com/

[6] R Core Team. R: A Language and Environment for Statistical Computing. Vienna, Austria. 2016. Retrieved from https://www.R-project.org/

[7] Andrews SR. FastQC: a quality control tool for high throughput sequence data. 2010. Retrieved from http://www.bioinformatics.babraham.ac.uk/projects/fastqc

[8] Bolger AM, Lohse M, Usadel B. Trimmomatic: A flexible trimmer for Illumina Sequence Data. *Bioinformatics*. 2014; btu170.

[9] Ewels P, Magnusson M, Lundin S, Käller M. MultiQC: summarize analysis results for multiple tools and samples in a single report. *Bioinformatics*. 2016; 32(19): 3047–3048.

[10] Patro R, Duggal G, Love MI, Irizarry RA, Kingsford C. Salmon provides fast and bias-aware quantification of transcript expression. *Nature Meth*. 2017; 14: 417-419.

[11] 53 Kalbfleisch TS, Rice ES, DePriest MS, Walenz BP, Hestand MS, Vermeesch JR, O'Connell BL, Fiddes IT, Vershinina AO, Saremi NF, Petersen JL, Finno CJ, Bellone RR, McCue ME, Brooks SA, Bailey E, Orlando L, Green RE, Miller DC, Antczak DF, MacLeod JN. Improved reference genome for the domestic horse increases assembly contiguity and composition. *Commun Biol*. 2018; 1(197).

[12] Love MI, Huber W, Anders S. Moderated estimation of fold change and dispersion for RNA-seq data with DESeq2. *Genome Biol.* 2014; 15(12).

[13] Love MI, Anders S, Kim V, Huber W. RNA-Seq workflow: gene-level exploratory analysis and differential expression*. F1000Res*. 2015; 4: 1070.

[14] Scott EY, Mansour T, Bellone RR, Brown Ct, Mienaltowski MJ, Penedo MC, Ross PJ, Valberg SJ, Murray JD, Finno CJ. Identification of long non-coding RNA in the horse transcriptome. *BMC Genomics*. 2017; 18(1):511.

[15] Mansour TA, Scott EY, Finno CJ, Bellone RR, Mienaltowski MJ, Pendo MC, Ross PJ, Valberg SJ, Murray JD, Brown CT. Tissue resolved, gene structure refined equine transcriptome. *BMC Genomics*. 2017; 18(1): 103.

[16] The Gene Ontology Consortium. Gene Ontology Annotations and Resources. *Nucleic Acids Res*. 2013; 41(Database Issue): D530-35.

[17] Krueger F. Trim Galore! 2012. Retrieved from http://www.bioinformatics.babraham.ac.uk/projects/trim_galore/

[18] Krueger F, Andrews SR. Bismark: a flexible aligner and methylation caller for Bisulfite-Seq applications. *Bioinformatics*. 2011; 27(11): 1571–1572.

[19] Akalin A, Kormaksson M, Li S, Garrett-Bakelman FE, Gigueroa ME, Melnick A, Mason CE. methylKit: a comprehensive R package for the analysis of genome-wide DNA methylation profiles. *Genome Biol.* 2012; 13(R87).

[20] Akalin A, Franke V, Vlahovicek K, Mason CE, Schubeler D. Genomation: a toolkit to summarize, annotate and visualize genomic intervals. *Bioinformatics*. 2015; 31: 1127–1129.

[21] Pechanec MY, Boyd TN, Baar K, Mienaltowski MJ. Adding exogenous biglycan or decorin improves tendon formation for equine peritenon and tendon proper cells in vitro. *BMC Musculoskelet Disord*. 2020; 21(1):627.

[22] Mienaltowski MJ, Huang L, Stromberg AJ, MacLeod JN. Differential gene expression associated with postnatal equine articular cartilage maturation. *BMC Musculoskelet Disord*. 2008;9:149.

[23] Vandesompele J, De Preter K, Pattyn F, Poppe B, Van Roy N, De Paepe A, Speleman F. Accurate normalization of real-time quantitative RT-PCR data by geometric averaging of multiple internal control genes. *Genome Biol.* 2002;3:RESEARCH0034.

[24] Ramakers C, Ruijter JM, Deprez RH, Moorman AF. Assumption-free analysis of quantitative real-time polymerase chain reaction (PCR) data. *Neurosci Lett.* 2003;339:62-6.

[25] Schefe JH, Lehmann KE, Buschmann IR, Unger T, Funke-Kaiser H. Quantitative real-time RT-PCR data analysis: current concepts and the novel "gene expression's CT difference" formula. *J Mol Med (Berl)*. 2006;84:901-10.
